# Analysis of US Food and Drug Administration new drug and biologic approvals, regulatory pathways, and review times, 1980–2022

**DOI:** 10.1038/s41598-024-53554-7

**Published:** 2024-02-09

**Authors:** Enrique Seoane-Vazquez, Rosa Rodriguez-Monguio, John H. Powers

**Affiliations:** 1 Department of Pharmaceutical Economic and Policy, Chapman University School of Pharmacy, Irvine, CA USA; 2 Economic Science Institute, Argyros School of Business and Economics, Chapman University, Orange, CA USA; 3 Department of Clinical Pharmacy, School of Pharmacy, University of California San Francisco, San Francisco, USA; 4 Medication Outcomes Center, University of California San Francisco, San Francisco, USA; 5 Philip R. Lee Institute for Health Policy Studies, University of California San Francisco, San Francisco, USA; 6 Department of Medicine, George Washington University School of Medicine, Washington, DC USA

**Keywords:** Drug regulation, Public health

## Abstract

U.S. laws enacted since 1983 have aimed to enhance the development and marketing of new pharmaceutical products. We thoroughly characterized all new molecular entities, therapeutic biologics, and gene and cell therapies approved by the US Food and Drug Administration (FDA) during the period 1980–2022 in the context of these laws and regulations. Throughout the study period, the FDA approved 1355 new pharmaceutical products. The median FDA review time decreased from 26.6 months prior to the Prescription Drug User Fee Act (1992), which authorized the FDA to collect fees from drug companies to 9.9 months after the Food and Drug Administration Safety and Innovation Act (2012), which created new designations that eliminated the requirement for evidence of added therapeutic benefit for FDA expedited drug review. The greatest increase in approvals occurred in antineoplastic and immunomodulating drugs, biologics, and orphan drugs. More than half of new drug approvals benefited from regulatory designations and pathways that did not require addressing unmet medical needs or demonstrating therapeutic benefit over available alternatives. The legislative goal of bringing more drugs to the market faster has been achieved. Further studies are needed to determine the therapeutic value to patients of new drugs approved using expedited approval pathways.

## Introduction

The mission of the FDA includes protecting and promoting public health by ensuring the safety and efficacy of drugs and biological products. The FDA is also responsible for advancing public health by “helping to speed innovation”^1^. The speed of drug development depends partly on acquiring the evidence required for marketing approval regarding the benefits and harms in specific patient populations.

The FDA regulatory review and approval processes reflect U.S. laws and FDA regulations, which have aimed to decrease FDA review time and increase the number of marketed new drugs (Table 1). The Prescription Drug User Fee Act (PDUFA) of 1992 authorized the FDA to collect fees from sponsor companies to expedite the review of drugs, representing a major milestone in FDA’s regulatory process with downstream effects on approval decisions. The FDA Safety and Innovation Act (FDASIA) of 2012 amended the fast-track designation and the accelerated approval pathway and removed the requirement of evidence of added therapeutic benefit over existing treatments. In addition, FDASIA created the breakthrough therapy designation, the rare pediatric disease priority review voucher, and the qualified infectious disease product designation (QIDP) for anti-infectives to treat serious or life-threatening diseases due to resistant or potentially resistant bacteria.

**Table 1:** FDA designations and expedited approval pathways, 1983–2022.

Program	Qualifying criteria	Main features
Orphan drug designation	A drug for a rare disease or condition that affects less than 200,000 persons in the U.S., or there is no reasonable expectation that the cost of developing the will be recovered from sales in the U.S. No added therapeutic benefit or disease severity required	7-Year market exclusivity to sponsors of approved orphan drugs, a tax credit of 25% percent of the cost of conducting human clinical trials, federal research grants for clinical testing, waiver of PDUFA user fees, FDA written recommendations for research study design, and use of open protocols for investigations
Orphan Drug Act (January 4, 1983)^8^		
Subpart E—Drugs intended to treat life-threatening and severely-debilitating illnesses	Drugs that are being studied for treating life-threatening or severely-debilitating diseases. No added therapeutic benefit required	Early consultation with the FDA in the drug development process. Expanded access to the investigational drug for treatment used by a large population (treatment protocol). Confirmatory Phase 4 studies to verify the drug risks, benefits, and optimal use. FDA monitors the progress of clinical trials and is involved in facilitating appropriate progress. FDA recognition that physicians and patients accept greater risks. Lower evidentiary approval standards^10^
21 CFR part 601, subpart E (October 21, 1988). Amended by FDASIA (July 9, 2012)^9^		
Subpart H—Accelerated approval of new drugs for serious or life-threatening illnesses	A drug that treats a serious or life-threatening disease and demonstrates an effect on a surrogate endpoint that is reasonably likely to predict a real clinical benefit. FDASIA removed the requirement of meaningful therapeutic benefit to patients over existing treatments	Approval based on a surrogate endpoint or on an effect on a clinical endpoint other than survival or irreversible morbidity. Potential approval with restrictions to assure safe use. Surrogate endpoints must be confirmed after marketing. Post-approval requirements may be satisfied by submission of evidence other than randomized clinical trials
21 CFR part 314, subpart H (December 11, 1992). Amended by FDASIA (July 9, 2012)^9^		
Priority review designation	A drug that treats a serious condition that is expected to provide a significant improvement in safety or effectiveness. No added therapeutic benefit or severity required for drugs designated as a qualified infectious disease product or submitted with a priority review voucher	Direct FDA’s overall attention and resources to expedite the assessment of priority review drugs. FDA's goal is to act on a priority review application within 6 months (compared to 10 months under standard review). Authorized the FDA to collect fees from companies to partially offset the cost of reviewing drugs for marketing approval. Between 1975 and 1992, priority review applied to drugs representing an important or modest therapeutic gain and did not have a time goal for review^11^
PDUFA (October 29, 1992, reauthorized six times, last time on September 30, 2022)^9^		
Fast track review designation	A drug that is intended to treat a serious condition and nonclinical or clinical data demonstrate the potential to address an unmet medical need. No added therapeutic benefit required for drugs with qualified infectious disease products designation	Actions to expedite regulatory review, eligibility for rolling review—FDA reviewing portions of the application before the sponsor submits the complete application-, and eligibility for accelerated approval and priority review
FDAMA (November 21, 1997). Amended by FDASIA (July 9, 2012)^9^		
Tropical disease, rare pediatric disease, and material threat medical countermeasure priority review vouchers	New drugs for prevention or treatment of a tropical disease, a serious or life-threatening rare disease or condition that affects individuals aged from birth to 18 years, or intended to diagnose, prevent, or treat diseases or conditions associated with chemical, biological, radiological, and nuclear threats, and emerging infectious diseases, and eligible for priority review (significant improvement)	The voucher can be used by the sponsor for a subsequent application that would not meet the requirements for a priority review or can be sold to another company
FDAAA (Sep 27, 2007). FDASIA (Jul. 9, 2012). 21st Century Cures Act (December 13, 2016)^12–14^		
Breakthrough therapy designation	A drug that is intended to treat a serious condition and preliminary clinical evidence indicate that the drug may demonstrate substantial improvement on a clinically significant endpoint(s) over available therapies	Fast-track designation features, intensive guidance on efficient drug development, and organizational commitment involving senior managers
FDASIA (July 9, 2012)^9^		
Qualified infectious disease product (QIDP) designation	A drug intended to treat serious or life-threatening infections caused by antibacterial or antifungal resistant pathogens or qualifying pathogens listed by the FDA. No added therapeutic benefit required for approval	5-year exclusivity extension (in addition to any other exclusivities), priority review designation, and fast track designation at the sponsor's request
FDASIA (July 9, 2012)^15^		
Limited population for antibacterial and antifungal drugs (LPAD) pathway	A drug intended to treat serious or life-threatening infections in a limited population of patients (a group of patients that is limited in a way that is clinically relevant to health care providers) to address unmet medical needs. No added therapeutic benefit required for approval	FDA advice to reduce drug development time and to conduct any additional studies required to gain approval for use in a broader population. FDA determination of safety and effectiveness considers the severity, rarity, or prevalence of the infection. FDA acceptance of greater uncertainty or higher risk. FDA may approve a drug based upon a conclusion of a positive benefit-risk balance in the limited population, even though insufficient data exist to conclude that there is a favorable benefit-risk profile in a broader population. Streamlined approaches including a single non-inferiority clinical trial, wider non-inferiority margins, and smaller or shorter clinical trials
21st Century Cures Act (December 13, 2016)^16^		
Regenerative medicine advanced therapy (RMAT) designation	Cell therapy, therapeutic tissue engineering, human cell and tissue products, and preliminary clinical evidence indicates that the drug has the potential to address unmet medical needs for serious or life-threatening diseases or conditions	All the benefits of the fast track and breakthrough therapy designation programs. Potential ways to support accelerated approval and satisfy post-approval requirements. Preliminary clinical evidence may be prospective clinical trials with a concurrent control, clinical investigations with historical controls, retrospective studies, or clinical case series, and include evidence from studies conducted outside of the U.S. Accelerated approval post-approval requirements fulfilled through the submission of clinical evidence, clinical studies, patient registries, or other sources of real-world evidence or post-approval monitoring of all patients treated prior to FDA approval
21st Century Cures Act (December 13, 2016)^17^		

FDASIA, FDA Safety and Innovation Act; PDUFA, Prescription Drug User Fee Act; FDAMA, FDA Modernization Act; FDAAA, Food and Drug Administration Amendments Act.

Previous studies have examined new drugs approved by the FDA during designated time frames^2–6^. However, there is a lack of up to date, comprehensive studies that assess the characteristics of all FDA-approved new drugs and biologics across therapeutic classes within the context of major regulations implemented in the US since 1980. Given the substantial public and private resources invested in the development of new therapies and the dynamic regulatory environment, it is crucial to assess the extent to which these laws and FDA regulatory actions have effectively accomplished their intended objective of advancing public health through drug approvals with evidence of addressing unmet patient needs and improving patient outcomes^7^. Therefore, we conducted a comprehensive assessment and characterization of all new molecular entities, therapeutic biologics, and gene and cell therapies approved by the FDA since 1980. Additionally, we analyzed the approval pathways and regulatory designations within the context of the legislative and regulatory landscape in the US.

## Methods

We collected the FDA regulatory information for all new active ingredients approved by the FDA for marketing in the U.S., including new molecular entities (NME)—a new drug never approved before by the FDA or marketed in the U.S.-, new therapeutic biologics, and gene and cell therapies in the period 1980 through 2022. We collected the application number, non-proprietary and brand names, FDA submission and approval dates, and the FDA regulatory designations and approval pathways. We extracted data for NMEs and therapeutic biologics from the Drugs@FDA, the Orange Book, and the Purple Book^18–20^. We extracted gene and cell therapies data from the list of Approved Cellular and Gene Therapy Products approved in the US available at the Centers for Biologic Evaluation and Research^21^. We also collected information about notices of FDA regulatory actions available at the Federal Register^22^ and the therapeutic class from the WHO Anatomical Therapeutic Chemical Classification System^23^ (Supplemental Table S1). We classified the drugs using the anatomical main group. We chose the therapeutic subgroup for systemic anti-infectives due to Congress approval of legislation incentivizing antibiotics and for diagnostic drugs because of their distinctions from products intended for treatment. We extracted all data through December 31, 2022. To ensure the reliability of the data, one investigator [ESV] was responsible for primary data extraction and placement into evidence tables and a second investigator [RRM] verified the data extraction and entry process. Data discrepancies were discussed and resolved by consensus.

The unit of analysis was the first application for each drug product. We calculated the FDA review time as the difference between the first new drug application (NDA) or biologic license application (BLA) submission and approval dates stated in FDA approval letters. We presented results by year of approval and three distinct periods defined by PDUFA (1992) and FDASIA (2012) enactment. Hence, the analysis was broken down by FDA approvals from 1980 to PDUFA enactment (1980-PDUFA), PDUFA to FDASIA enactment (PDUFA-FDASIA), and thereafter (FDASIA-2022).

## Results

### FDA approved new molecular entities, biologics and gene and cell therapy

**Figure 1:**
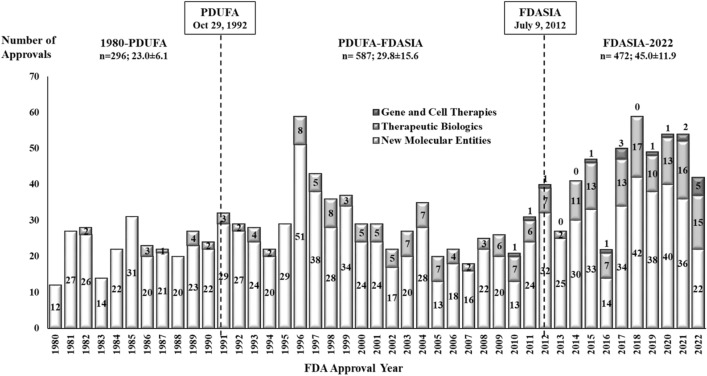
FDA New Drug Approvals by Type of Product, 1980–2022. Annual FDA approvals of new molecular entities, therapeutic biologics, and gene and cell therapies from 1980 to 2022. The figure provides the number of approvals and the annual average ± standard deviation of new drugs approved during the periods defined by the Prescription Drug User Fee Act of 1992 (PDUFA) and the Food and Drug Administration Safety and Innovation Act of 2012 (FDASIA).

In the period 1980–2022, the FDA approved 1355 new drugs, with an annual drug approval average ± standard deviation of 31.5 ± 12.0 drugs (Table 2). The annual average number of approvals increased from 23.1 ± 6.1 (1980-PDUFA) to 29.8 ± 15.6 (PDUFA-FDASIA) and 45.0 ± 11.9 (FDASIA-2022) (Fig. 1). FDA approvals included 1103 (81.4%) NME, 235 (17.3%) therapeutic biologics, and 17 (1.3%) gene and cell therapies. The annual average number of approved NME increased from 21.8 ± 6.4 (1980-PDUFA) to 24.7 ± 15.3 (PDUFA-FDASIA) and 32.1 ± 9.7 in FDASIA-2022. Whereas the annual average number of approved therapeutic biologics increased from 1.2 ± 1.0 (1980-PDUFA) to 5.0 ± 1.8 (PDUFA-FDASIA) and 11.5 ± 4.4 in FDASIA-2022. The FDA approved 17 gene and cell therapies in the period 2010 through 2022.

**Table 2:** FDA new drug approvals by regulatory period and therapeutic class, 1980–2022.

Therapeutic class	1980-PDUFA (12.8 years)		PDUFA-FDASIA (19.7 years)		FDASIA-2022 (10.5 years)		Total Approvals (43.0 years)	
	Approvals (%)	Average ± SD	Approvals (%)	Average ± SD	Approvals (%)	Average ± SD	Approvals (%)	Average ± SD
Antineoplastic and immunomodulating agents	26 (8.8%)	2.0 ± 1.7	111 (18.9%)	5.6 ± 2.0	169 (35.8%)	16.1 ± 5.9	306 (22.6%)	7.1 ± 6.2
Nervous system	30 (10.1%)	2.3 ± 1.7	80 (13.6%)	4.1 ± 3.5	47 (10.0%)	4.5 ± 2.6	157 (11.6%)	3.7 ± 2.3
Alimentary tract and metabolism	22 (7.4%)	1.7 ± 1.3	59 (10.1%)	3.0 ± 1.7	53 (11.2%)	5.1 ± 2.8	134 (9.9%)	3.1 ± 2.1
Cardiovascular system	54 (18.2%)	4.2 ± 2.3	45 (7.7%)	2.3 ± 2.6	19 (4.0%)	1.8 ± 1.5	118 (8.7%)	2.7 ± 2.0
Diagnostic drugs	28 (9.5%)	2.2 ± 1.8	39 (6.6%)	2.0 ± 4.0	20 (4.2%)	1.9 ± 1.2	87 (6.4%)	2.0 ± 1.9
Antibacterials for systemic use	38 (12.8%)	3.0 ± 1.3	24 (4.1%)	1.2 ± 1.3	15 (3.2%)	1.4 ± 1.7	77 (5.7%)	1.8 ± 1.6
Antivirals for systemic use	7 (2.4%)	0.5 ± 0.8	34 (5.8%)	1.7 ± 1.3	25 (5.3%)	2.4 ± 1.3	66 (4.9%)	1.5 ± 1.3
Blood and blood forming organs	5 (1.7%)	0.4 ± 0.7	37 (6.3%)	1.9 ± 1.1	21 (4.4%)	2.0 ± 1.6	63 (4.6%)	1.5 ± 1.3
Musculo-skeletal system	20 (6.8%)	1.6 ± 1.0	22 (3.7%)	1.1 ± 1.6	14 (3.0%)	1.3 ± 1.3	56 (4.1%)	1.3 ± 1.2
Dermatologicals	17 (5.7%)	1.3 ± 1.5	19 (3.2%)	1.0 ± 1.5	17 (3.6%)	1.6 ± 1.0	53 (3.9%)	1.2 ± 1.2
Respiratory system	9 (3.0%)	0.7 ± 0.8	24 (4.1%)	1.2 ± 1.6	13 (2.8%)	1.2 ± 0.8	46 (3.4%)	1.1 ± 1.0
Sensory organs	7 (2.4%)	0.5 ± 0.7	28 (4.8%)	1.4 ± 0.9	12 (2.5%)	1.1 ± 1.0	47 (3.5%)	1.1 ± 0.9
Genito urinary system and sex hormones	8 (2.7%)	0.6 ± 0.7	21 (3.6%)	1.1 ± 1.6	10 (2.1%)	1.0 ± 0.9	39 (2.9%)	0.9 ± 1.0
Antiparasitic products, insecticides and repellents	10 (3.4%)	0.8 ± 0.8	9 (1.5%)	0.5 ± 1.4	9 (1.9%)	0.9 ± 1.0	28 (2.1%)	0.7 ± 0.8
Systemic hormonal preparations, excl. sex hormones & insulins	6 (2.0%)	0.5 ± 0.5	9 (1.5%)	0.5 ± 0.7	9 (1.9%)	0.9 ± 1.1	24 (1.8%)	0.6 ± 0.7
Other antiinfectives for systemic use	4 (1.4%)	0.3 ± 0.5	8 (1.4%)	0.4 ± 1.4	8 (1.7%)	0.8 ± 1.3	20 (1.5%)	0.5 ± 0.7
All other therapeutic products	5 (1.7%)	0.4 ± 0.9	18 (3.1%)	0.9 ± 1.2	11 (2.3%)	1.0 ± 1.4	34 (2.5%)	0.8 ± 1.2
Total	296 (100%)	23.1 ± 6.1	587 (100%)	29.8 ± 15.6	472 (100%)	45.0 ± 11.9	1355 (100%)	31.5 ± 12.0

In the study period, antineoplastic and immunomodulating agents (306, 22.6% of total approvals) had the greatest number of approvals among all therapeutic classes (Table 1). Cardiovascular system drugs (54, 18.2%) had the greatest number of approvals in the period from 1980 to the enactment of PDUFA. Since 1992, antineoplastic and immunomodulating agents continued to dominate with the greatest number of approvals, both during the PDUFA-FDASIA period (111, 18.9%) and the FDASIA-2022 period (169, 35.8%) (Table 2).

### FDA designations and expedited approval pathways

The FDA approved over half of the new drugs 788 (58.2%) using at least one designation or expedited approval pathway during the study period (Table 3) with an annual average of 18.3 ± 10.7 new drugs. The annual average number of drugs approved through an FDA designation or expedited review increased from 11.3 ± 4.0 (145, 49.0%) in the period from 1980 to the enactment of PDUFA, to 14.8 ± 7.4 (292, 49.7%) in the PDUFA-FDASIA period, and 33.5 ± 10.4 (351, 74.4%) in the FDASIA-2022 period.

**Table 3:** FDA designations and regulatory approval pathways for new drugs, 1980–2022.

Therapeutic class	Approvals	Orphan drug designation at first approval	Priority review	Accelerated approval	Fast track	Breakthrough therapy	Qualified infectious disease product	Any expedited review designation or process
Antineoplastic & immunomodulating agents	306	181 (59.7%)	224 (73.2%)	89 (30.1%)	88 (34.9%)	62 (36.7%)	–	261 (85.3%)
Nervous system	157	27 (18.4%)	47 (29.9%)	1 (0.7%)	11 (11.7%)	5 (10.6%)	–	58 (36.9%)
Alimentary tract and metabolism	134	47 (35.9%)	69 (51.5%)	3 (2.6%)	24 (25.5%)	13 (25.0%)	2 (100.0%)	74 (55.2%)
Cardiovascular system	118	16 (15.2%)	34 (28.8%)	2 (2.3%)	7 (17.1%)	2 (10.5%)	–	41 (34.7%)
Diagnostic drugs	87	11 (13.9%)	33 (37.9%)	0 (0.0%)	2 (5.7%)	0 (0.0%)	–	36 (41.4%)
Antibacterials for systemic use	77	0 (0.0%)	31 (40.3%)	1 (1.9%)	11 (39.3%)	0 (0.0%)	14 (93.3%)	31 (40.3%)
Antivirals for systemic use	66	8 (12.1%)	55 (83.3%)	20 (31.3%)	24 (51.1%)	12 (48.0%)	–	58 (87.9%)
Blood and blood forming organs	63	25 (39.7%)	40 (63.5%)	3 (4.9%)	13 (28.3%)	6 (28.6%)	–	46 (73.0%)
Musculo–skeletal system	56	16 (31.4%)	22 (39.3%)	5 (11.4%)	7 (24.1%)	2 (14.3%)	–	26 (46.4%)
Dermatologicals	53	4 (8.5%)	11 (20.8%)	0 (0.0%)	2 (7.4%)	1 (5.9%)	–	13 (24.5%)
Respiratory system	46	11 (25.0%)	14 (30.4%)	0 (0.0%)	6 (25.0%)	3 (23.1%)	–	18 (39.1%)
Sensory organs	47	5 (10.9%)	30 (63.8%)	0 (0.0%)	3 (10.0%)	3 (25.0%)	–	31 (66.0%)
Genito urinary system and sex hormones	39	2 (5.4%)	7 (17.9%)	0 (0.0%)	1 (4.0%)	0 (0.0%)	–	8 (20.5%)
Antiparasitic products, insecticides & repellents	28	18 (78.3%)	23 (82.1%)	2 (9.5%)	4 (28.6%)	2 (22.2%)	–	24 (85.7%)
Systemic hormonal preparations, excluding sex hormones & insulins	24	12 (50.0%)	12 (50.0%)	0 (0.0%)	3 (17.6%)	0 (0.0%)	–	15 (62.5%)
Other antiinfectives for systemic use	20	9 (47.4%)	17 (85.0%)	2 (11.1%)	7 (46.7%)	1 (12.5%)	3 (37.5%)	18 (90.0%)
All other therapeutic products	34	20 (60.6%)	26 (76.5%)	5 (15.2%)	8 (34.8%)	4 (36.4%)	–	30 (88.2%)
Total	1355	412 (32.0%)	695 (51.3%)	133 (11.4%)	221 (26.3%)	116 (24.7%)	19 (76.0%)	788 (58.2%)

The percentages refer to drugs approved after the implementation of the respective designations and procedures.

The FDA granted priority review designation to 695 (51.3%) drugs, including 15 (1.1% of total approvals) drugs approved using a priority review voucher and 19 (1.4%) drugs granted the Qualified Infectious Disease Product Designation (QIDP). Since 1992, the FDA approved 133 (11.4%) drugs through the accelerated approval pathway using surrogate endpoints. In addition, the FDA approved 221 (26.2%) drugs using the fast-track review designation and granted breakthrough therapy designation to 116 (24.7%) new drugs (Table 3).

**Figure 2:**
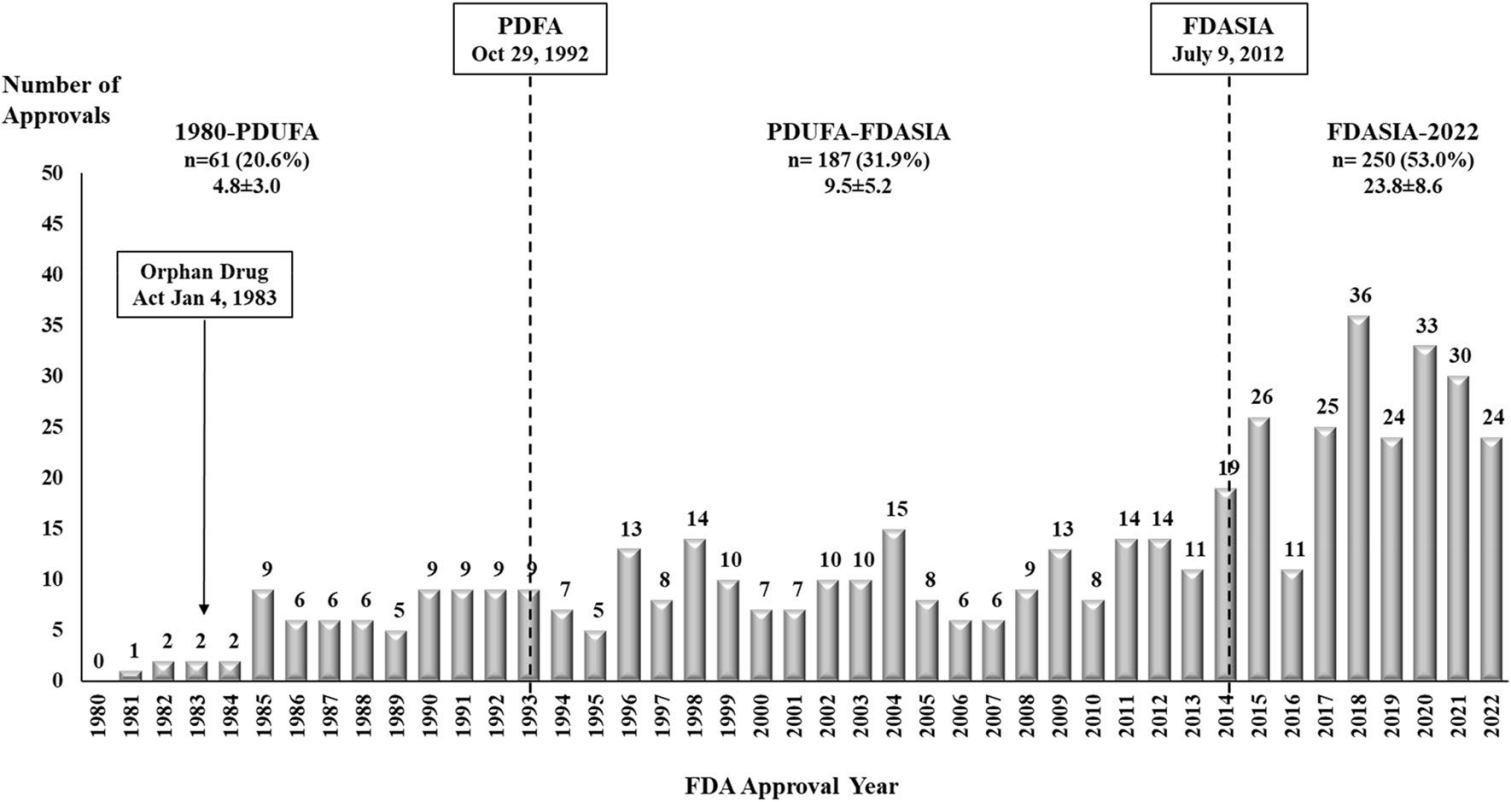
New Drugs with at Least One FDA Orphan Drug Approval, 1980–2022. New drugs approved in 1980–2022 with a least one approved orphan indication based on the year of their initial FDA approval. The figure presents the number of drugs and the annual average ± standard deviation of drugs with orphan designation during the periods defined by PDUFA and FDASIA. The FDA holds the authority to approve new drug indications with orphan designation during or after the initial approval.

The FDA granted at least one orphan designation to 498 (36.8%) drugs during the study period, including 412 new drugs with at least one orphan designation at first approval. The average number of approvals with at least one orphan designation increased from 4.8 ± 3.0 (61, 20.6%) in the period from 1983 to the enactment of PDUFA, to 9.5 ± 5.2 (187, 31.9%) in the PDUFA-FDASIA period, and 23.8 ± 7.9 (250, 53.0%) in the FDASIA-2022 period (Fig. 2).

Antineoplastic and immunomodulating agents was the therapeutic class with the greatest percentage of approvals overall and with orphan drug designations (181, 59.7%), priority review designations (224 73.2%), accelerated approvals (89, 30.1%), fast track designations (88, 34.9%), and breakthrough therapy designations (62, 36.7%) (Table 3).

Since the enactment of the QIDP designation in 2012, 19 (76.0%) out of 25 of the eligible antibacterials and antifungals were approved by the FDA using this designation. Additionally, the Limited Population Approval Pathway (LPAD) for antibacterial and antifungal drugs was used for two drugs: pretomanid and amikacin liposome inhalation suspensions. The FDA approved pretomanid on August 14, 2019, for the treatment of drug-resistant tuberculosis, while amikacin liposome inhalation suspension (new formulation) received FDA approval on September 28, 2018, for refractory lung infections caused by Mycobacterium avium complex. Moreover, the FDA granted the Regenerative Medicine Advanced Therapy (RMAT) designation to two cell therapies: allogeneic cultured keratinocytes and dermal fibroblasts in murine collagen-dsat, as well as allogeneic processed thymus tissue–agdc. One gene therapy, lisocabtagene maraleucel, also received the RMAT designation.

### FDA approval review time

**Figure 3:**
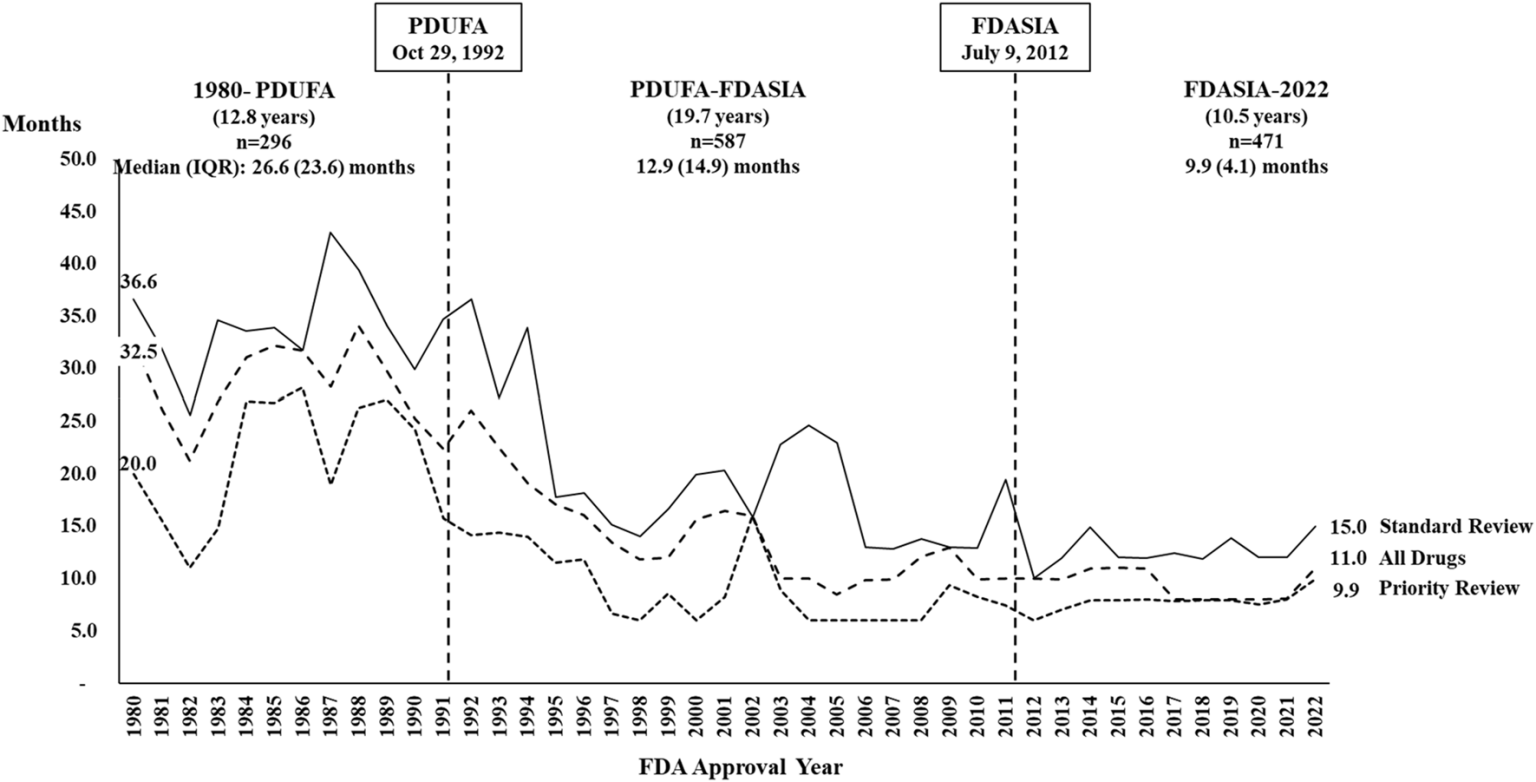
FDA New Drug Approvals Median Review Time, 1980–2022. Annual median and interquartile range (IQR) of FDA review time for new drugs approved between 1980 and 2022. The trends are presented for standard review, priority review, and the total for all drugs. The figure includes the number of drugs and the annual median and IQR of the FDA review time for new drugs during the periods defined by PDUFA and FDASIA.

In the period 1980 through 2022, the median (interquartile range -IQR) FDA review time for new therapeutics was 12.0 (16.3) months (Table 4). Specifically, the median (IQR) review time for priority review drugs was 8.1 (9.0) months, whereas for standard review drugs, it was 18.2 (19.9) months. Over time, the median FDA review time significantly decreased from 26.6 (23.6) months in the pre-PDUFA period, to 12.9 (14.9) in the PDUDA-FDASIA period, and 9.9 (4.1) months in the FDASIA-2022 period (Fig. 3). The review time varied by therapeutic class (Supplemental Table S2).

**Table 4:** FDA regulatory approval review time in months by therapeutic class and PDUFA Period, 1980–2022.

Therapeutic class	Pre-PDUFA	PDUFA (Oct 12, 1992)	PDUFA II (Nov 21, 1997)	PDUFA III (June 12, 2002)	PDUFA IV (Sept 27, 2007)	PDUFA V (July 9, 2012)	PDUFA VI (Aug 18, 2017)	Total
Alimentary tract and metabolism	20.4 (19.1)	13.2 (7.5)	8.9 (6.9)	6.0 (3.5)	9.0 (3.9)	7.9 (5.1)	7.9 (4.4)	8.1 (6.0)
All other therapeutic products	39.0 (25.8)	19.3 (18.3)	17.0 (9.8)	15.6 (18.4)	16.9 (16.4)	12.7 (13.0)	12.0 (5.9)	17.4 (20.4)
Antibacterials for systemic use	22.6 (21.8)	15.0 (6.9)	14.3 (12.0)	10.0 (20.0)	11.4 (12.1)	12.0 (1.9)	9.0 (3.8)	12.0 (10.5)
Antineoplastic and immunomodulating agents	35.1 (24.1)	27.0 (20.6)	13.9 (6.4)	11.3 (13.7)	12.0 (14.4)	9.7 (4.0)	8.0 (6.4)	24.2 (26.5)
Antiparasitic products, insecticides and repellents	32.0 (21.7)	31.4 (14.3)	21.2 (15.8)	38.4 (18.2)	15.4 (10.8)	11.5 (7.2)	13.8 (9.9)	26.1 (20.5)
Antivirals for systemic use	23.3 (15.1)	26.2 (11.5)	11.7 (3.4)	24.2 (33.9)	10.0 (11.4)	8.0 (0.1)	8.0 (0.0)	18.7 (16.2)
Blood and blood forming organs	12.2 (27.6)	5.1 (6.5)	5.9 (1.5)	6.0 (1.5)	6.0 (0.1)	8.0 (2.2)	8.0 (3.0)	7.6 (4.0)
Cardiovascular system	18.9 (5.1)	16.0 (10.6)	14.1 (10.7)	9.0 (15.6)	16.4 (9.5)	12.0 (3.6)	7.9 (3.1)	12.2 (10.8)
Dermatologicals	34.5 (33.3)	21.4 (24.9)	12.8 (7.9)	10.0 (0.0)	17.0 (7.0)	11.5 (3.3)	11.1 (11.2)	16.9 (21.6)
Diagnostic drugs	17.1 (12.8)	19.7 (4.8)	12.0 (11.4)	13.7 (5.1)	18.8 (8.8)	11.3 (0.3)	12.3 (5.2)	14.3 (11.0)
Genito urinary system and sex hormones	41.0 (18.1)	28.7 (58.2)	32.6 (12.1)	25.6 (13.3)	24.9 (31.1)	11.9 (2.8)	7.5 (4.5)	25.6 (29.0)
Musculo-skeletal system	15.9 (10.7)	12.0 (7.6)	6.9 (6.7)	6.0 (3.9)	9.9 (2.2)	7.8 (5.2)	8.0 (1.9)	9.9 (7.4)
Nervous system	20.7 (14.8)	23.3 (25.7)	12.0 (11.5)	23.9 (4.9)	10.0 (0.1)	11.0 (16.4)	12.0 (2.4)	14.9 (13.3)
Other antiinfectives for systemic use	13.0 (15.1)	7.4 (1.6)	–	7.9 (2.1)	21.8 (7.3)	11.0 (0.0)	8.0 (2.1)	9.7 (8.2)
Respiratory system	27.7 (12.9)	–	12.2 (6.4)	8.6 (9.2)	17.4 (0.0)	13.0 (5.1)	12.0 (37.4)	14.9 (11.0)
Sensory organs	14.2 (9.0)	11.2 (0.0)	6.0 (3.0)	33.8 (12.9)	–	10.9 (4.0)	8.0 (2.1)	10.9 (10.0)
Systemic hormonal preparations, excluding sex hormones & insulins	12.3 (8.2)	30.5 (23.9)	13.8 (6.9)	6.4 (24.8)	19.2 (13.2)	9.9 (26.2)	12.0 (17.6)	12.0 (21.7)
Standard review drugs	33.5 (23.3)	22.9 (20.5)	16.2 (11.7)	17.8 (19.0)	13.0 (13.7)	12.0 (3.7)	12.0 (4.9)	18.2 (19.9)
Priority review drugs	21.6 (18.5)	12.1 (14.8)	6.4 (6.4)	6.0 (3.1)	7.1 (4.3)	8.0 (4.9)	8.0 (1.0)	8.1 (9.0)
Total	26.6 (23.6)	18.5 (18.1)	12.2 (11.7)	10.0 (18.6)	10.0 (10.8)	10.6 (4.1)	8.1 (4.2)	12.0 (16.3)

## Discussion

This study comprehensively characterizes all FDA-approved new molecular entities, therapeutic biologics, and gene and cell therapies by therapeutic class and regulatory approval pathway since the first publication of the electronic version of the Approved Drug Products with Therapeutic Equivalence Evaluations, commonly known as the Orange Book, in 1980. Consistent with previous ad-hoc studies^3–6,24^, our study provides evidence that legislation enacted by U.S. Congress and FDA regulations implemented over the past four decades have effectively contributed to the increased number of pharmaceutical products approved for marketing in the US. Specifically, we found that over half of the pharmaceutical products obtained FDA approval using at least one FDA designation, particularly the orphan drug designation, or expedited approval pathway. Since the enactment of PDUFA in 1992, the average number of approvals using the priority review designation more than doubled, and more than one in ten of the new drug approvals used accelerated approval pathways based on a surrogate endpoint. Since 1997, over one-fourth of the new drug approvals have been approved using the fast-track designation, where no evidence demonstrating the potential to address an unmet medical need is required. Lastly, since the enactment of FDASIA in 2012, more than one in five new drug approvals used the breakthrough therapy designation based on preliminary clinical evidence of potential improvement over available therapies. This increase in approvals was primarily driven by therapeutic biologics with a notable emphasis on those indicated for cancer conditions.

We also found a significant reduction in the FDA regulatory review time after the enactment of PDUFA in 1992, a finding that is consistent with a previous study^5^. A study reviewing drug approval applications in the period 2000–2011 concluded that the FDA completed 90% of the priority reviews within the 6 months of PDUFA established timeframe^25^. We found a reduction in the median review time from 27.1 months before PDUFA to 9.9 months after FDASIA. Greater reductions in the FDA review time could have been achieved if preventable sponsor deficiencies during the drug development process (populations, endpoints, adverse events, optimal doses, and labeling errors) had not occurred^25^.

In the late 1980s, insufficient federal funding adversely impacted FDA operations, contributing to a backlog of drug applications ^26^. PDUFA provided the FDA with additional resources to expedite new drug application reviews, resulting in a significant reduction in FDA review times. The acceptance of industry fees has raised concerns about the independence and transparency of FDA when regulating the industry^26,27^.

The Kefauver–Harris Amendments of 1962 required drug sponsor companies to provide evidence of safety and effectiveness derived from two adequate and well-controlled clinical studies before a new drug can enter the US market, adhering to the basic scientific concept of confirmation of evidence as any study might give erroneous results by chance alone, bias or lack of data integrity The evidence should be sufficient for the FDA to determine that the drug’s benefits outweigh its risks to patients^28^. The 1962 Amendments were appended by several regulations, including the Orphan Drug Act (1983), the PDUFA (1992, reauthorized six times), and the 21st Century Cures Act (2016). The FDA interpreted these regulations to allow what it terms “flexibility” regarding various factors including the endpoints used as the basis for approval and the number, type, and characteristics of clinical studies used to demonstrate safety and effectiveness at regulatory review, particularly for drugs indicated for rare, serious, and life-threatening diseases, and unmet medical needs. However, none of the legislative changes altered the requirements for “substantial evidence” from “adequate and well-controlled investigations”. After the enactment of FDAMA (1997), the quantity of information was altered as evidence derived from one adequate and well-controlled clinical trial plus other “confirmatory evidence” (not defined in law or regulation) may be sufficient for drug approval. The FDA has acknowledged that requiring only one clinical trial or using non-randomized trial designs may result in design flaws, random error, and biases in study conduct and results, potentially leading to an erroneous conclusion that a drug is effective^10^. An FDA review of 22 drugs found that Phase II clinical trial results can inaccurately predict the safety and effectiveness of medical products across a wide range of diseases and patient populations^29^.

The expedited approval pathways also resulted in significant changes in the outcomes used as endpoints evaluated in clinical trials^30^. We found that more than one in ten of the drug products approved after the enactment of FDASIA (2012) used subpart H surrogate endpoints which are indirect measures of patient outcomes judged by FDA as “reasonably likely” to predict direct patient benefits. The FDA acknowledged that changes implemented in study endpoints and evidence required for approval affect the drug benefits and risks. According to the FDA, the use of surrogate endpoints “almost always introduces some uncertainty into the risk–benefit assessment, because a clinical benefit is not measured directly and the quantitative relation of the effect on the surrogate to the clinical effect is rarely known”^10^. Furthermore, adverse effects are often direct measures of patient’s health (e.g., nausea, headache) while the “benefits” are indirect measures whose relationship to patient’s health often is unclear. Whenever a new drug is approved for marketing without robust evidence of a direct patient benefit, the sponsor company is required to continue assessing drug safety and effectiveness after approval to confirm direct patient benefits. Hence, there might be a risk of approving drugs for marketing that would otherwise be considered investigational medical products that could be ineffective or cause unexpected serious adverse effects. In addition, the approval of new drugs without reliable confirmatory evidence of their safety and effectiveness transfers the burden of the decision about the risk-benefits trade-off to clinicians and patients. The use of expedited approval pathways of unclear therapeutic value or for prevalent diseases and conditions represents a departure from the intended purpose of the expedited FDA review process^11^, initially intended for cancer and AIDS treatments^27^. Previous studies found a decrease in the quality of the evidence derived from clinical trials^31,32^ and in the number of pivotal trials used for approval of new drugs^32^. Additionally, there are significant delays in confirmatory trials for drug applications granted FDA’s expedited approval^34^.

The FDA granted marketing approval to a growing number of drugs with orphan designation in the study period. Almost half of the FDA-approved drugs in 2016–2022 had at least one orphan designation, which is more than in the first 20 years after the passage of the Orphan Drug Act in 1983. Drugs qualifying for the orphan drug designation affect less than 200,000 persons in the U.S., even if the disease is prevalent worldwide (e.g., tuberculosis)^35–37^. Long-term trends indicate an increasing orphanization of drug development in the U.S.^38^. The Orphan Drug Act established several incentives for the development of drugs for rare diseases, including public funding, tax credits, waivers of filing fees, and 7-year market exclusivity^36,38,39^. The orphan drug designation does not require demonstration of added patient benefit. In addition, drugs for rare diseases and conditions often qualify for expedited designations and regulatory pathways, flexibility in the design of studies required to demonstrate the effectiveness and to establish safety, and a shorter development time than other drugs^40^.

Providing patients with unmet medical needs faster access to drugs to treat serious and life-threatening diseases has been argued as a factor for expediting the drug development and approval processes^30^. Nevertheless, new drug sponsors do not need to provide evidence of added patient benefits to qualify for several of the implemented designations and expedited regulatory procedures implemented in the U.S., including orphan drug designation, accelerated approval, qualified infectious disease product designation, and drugs approved using priority review vouchers. Furthermore, the FDA’s operational definition of “severe disease” and “unmet medical need” may open the application of expedited regulations and procedures to an increasing number of pharmaceutical products. According to the FDA, a serious disease or condition is expected to be associated with morbidity that has a substantial impact on day-to-day functioning. The FDA also considers as serious a disease “a matter of clinical judgment” based on the likelihood that the disease, “if left untreated, will progress from a less severe condition to a more serious one” without considering whether other therapies are already approved for that disease and patient population thus, making the “left untreated” criterion clinically irrelevant^9^. The FDA’s definition of unmet medical need, “a condition whose treatment or diagnosis is not addressed adequately by available therapy,” is also debatable. An effective off-label treatment, such as the use of an antibiotic combination for drug-resistant bacteria, would not be considered as “available therapy” because it has not been approved by the FDA, again divorcing the consideration from clinical practice evidence. Lastly, the FDA concluded that “a drug that is not shown to provide a direct efficacy or safety advantage over an available therapy may nonetheless provide an advantage that would be of sufficient public health benefit to qualify as meeting an unmet medical need.” This definition seems both vague and broad. It is unclear how such a pharmaceutical product would improve patient outcomes in the absence of confirmatory evidence as the hypothesis of “public health benefit” remains untested and every drug has “potential” to address unmet needs prior to evaluation in adequate and well-controlled studies as required by law.

This lack of robust evidence of patient benefits is exemplified in the case of qualified infectious disease products (QIDP). The FDA can approve a new antibiotic without added clinical benefit for an “unmet medical need” without evidence demonstrating added benefits for those patients, as the antibiotic may be approved based on clinical trials demonstrating non-inferiority in patients who have already marketed effective therapeutic alternatives. The FDA claims that the benefit of new QIDP anti-infectives is based on the notion that some patients fail to respond to the available therapy or by having a novel mechanism of action that “could benefit patients who no longer respond to available therapy.” These assumptions are based on untested hypotheses since there is a lack of evidence that the new QIDP drugs improve patient outcomes when available therapies fail^9^. Although the FDASIA does not provide a different standard for approval, the FDA has approved drugs via QIDP in studies without a stated hypothesis or appropriate use of inferential statistics (two criteria for “substantial evidence” required in FDA’s regulations) or demonstrated evidence of better effectiveness than existing therapeutic alternatives to qualify for priority review and fast track designation and to be granted 5 years of market exclusivity in addition to any exclusivity granted upon approval. Hence, new systemic antibiotics have been marketed at a higher price without generic competition in the absence of demonstrated added patient benefits^41,42^.

This study has some limitations. This study did not assess the post-approval assessment of clinical benefits of drugs approved by the FDA using designations and expedited review processes. The study did not assess either advances in scientific techniques and knowledge, the emergence of healthcare technologies, changes in healthcare systems, and other macroeconomic trends that may have altered incentives for new drug development. Certain biologic products, including blood, vaccines, and allergenics were not included. Study findings should be interpreted in the context of the laws and regulations implemented during the study period, notably PDUFA and FDASIA. It was beyond the scope of this study to evaluate whether new drug approvals improved patient outcomes or offered therapeutic gains for unmet medical needs once introduced into clinical practice. Study data included the first FDA application for NMEs and new biologic applications. An NME or new therapeutic biologic does not necessarily add value compared to available therapies. Conversely, a new approval of a drug already marketed may represent an improvement over the available alternatives. However, it is unlikely that including secondary approvals will change the overall trends and relationships observed in this study. Given the public and private resources expended in developing new therapies, it is important to understand better the safety and efficacy evidence required for the development and approval of pharmaceutical products. We plan on performing these analyses as the next step in our research.

## Conclusions

During the period 1980–2022, there was a substantial increase in the number of marketing approvals of new drug products, particularly biologics, with the majority being antineoplastic and immunomodulating agents. A significant proportion of the newly approved drugs were granted approval through designations and expedited review procedures, which do not require the demonstration of addressing unmet medical needs or providing superior patient benefits compared to existing marketed alternatives. Throughout the study period, the legislative objective of bringing more drugs to the US market more quickly has been accomplished; however, the regulatory basis for the quality of evidence for approval has lessened and not kept pace with the speed of approvals. Whether the new drugs approved via expedited pathways have enhanced patient outcomes or provided therapeutic advantages for unmet medical needs once introduced into clinical practice warrants further research.

### Supplementary Information

Supplementary Table 1.

Supplementary Table 2.

## Data Availability

The data used for the study are available at the FDA website Drugs@FDA https://www.fda.gov/drugs/drug-approvals-and-databases/about-drugsfda.
