# Supplementary material for: Analysis of US Food and Drug Administration new drug and biologic approvals, regulatory pathways, and review times, 1980–2022
Source: Sci Rep. 2024 Feb 9;14:3325. doi: 10.1038/s41598-024-53554-7 (PMC10858128; doi:10.1038/s41598-024-53554-7)
Supplement: Supplementary file 1 — Supplementary Table 1. [file 41598_2024_53554_MOESM1_ESM.docx]

S1 Table. FDA Data Sources

|  | **Pharmaceutical products included** | **Source** |
| --- | --- | --- |
| **Approval Databases** | | |
| Drugs@FDA: FDA-Approved Drugs | Drugs and Therapeutic Biologics | <https://www.accessdata.fda.gov/scripts/cder/daf/index.cfm> |
| Approved Drug Products with Therapeutic Equivalence Evaluations – Orange Book- | Drugs | <https://www.fda.gov/drugs/drug-approvals-and-databases/approved-drug-products-therapeutic-equivalence-evaluations-orange-book> |
| Purple Book Database of Licensed Biological Products | Biologics | [https://purplebooksearch.fda.gov](https://purplebooksearch.fda.gov/) |
| Approved Cellular and Gene Therapy Products | Cellular and Gene Therapy Products | <https://www.fda.gov/vaccines-blood-biologics/cellular-gene-therapy-products/approved-cellular-and-gene-therapy-products> |
| **Annual Reports** | | |
| New Drugs at FDA: CDER's New Molecular Entities and New Therapeutic Biological Products | Drugs and Therapeutic Biologics | <https://www.fda.gov/drugs/development-approval-process-drugs/new-drugs-fda-cders-new-molecular-entities-and-new-therapeutic-biological-products> |
| **Designations and Regulatory Approval Pathways** | | |
| Orphan Drug Designations and Approvals | Drugs and Biologics | <https://www.accessdata.fda.gov/scripts/opdlisting/oopd> |
| Priority New Drug Applications and Biologic License Applications | Drugs and Biologics | <https://www.fda.gov/drugs/nda-and-bla-approvals/priority-nda-and-bla-approvals> |
| Breakthrough Therapy Approvals | Drugs and Biologics | <https://www.fda.gov/drugs/nda-and-bla-approvals/breakthrough-therapy-approvals> |
| Fast Track Approvals | Drugs and Biologics | <https://www.fda.gov/drugs/nda-and-bla-approvals/fast-track-approvals> |
| Accelerated Approvals | Drugs and Biologics | <https://www.fda.gov/drugs/nda-and-bla-approvals/accelerated-approvals> |
