# Supplementary material for: Analysis of US Food and Drug Administration new drug and biologic approvals, regulatory pathways, and review times, 1980–2022
Source: Sci Rep. 2024 Feb 9;14:3325. doi: 10.1038/s41598-024-53554-7 (PMC10858128; doi:10.1038/s41598-024-53554-7)
Supplement: Supplementary file 2 — Supplementary Table 2. [file 41598_2024_53554_MOESM2_ESM.docx]

S2 Table. FDA Regulatory Approval Review Time in months by Therapeutic Class and PDUFA Period, 1980-2022

| **Therapeutic Class Median (Interquartile Range)** | **Pre-PDUFA** | **PDUFA (Oct 12, 1992)** | **PDUFA II (Nov 21, 1997)** | **PDUFA III (June 12, 2002)** | **PDUFA IV (Sept 27, 2007)** | **PDUFA V (July 9, 2012)** | **PDUFA VI (Aug 18, 2017)** | **Total** |
| --- | --- | --- | --- | --- | --- | --- | --- | --- |
| Alimentary Tract and Metabolism | 20.4 (19.1) | 13.2 (7.5) | 8.9 (6.9) | 6.0 (3.5) | 9.0 (3.9) | 7.9 (5.1) | 7.9 (4.4) | 8.1 (6.0) |
| All Other Therapeutic Products | 39.0 (25.8) | 19.3 (18.3) | 17.0 (9.8) | 15.6 (18.4) | 16.9 (16.4) | 12.7 (13.0) | 12.0 (5.9) | 17.4 (20.4) |
| Antibacterials for Systemic Use | 22.6 (21.8) | 15.0 (6.9) | 14.3 (12.0) | 10.0 (20.0) | 11.4 (12.1) | 12.0 (1.9) | 9.0 (3.8) | 12.0 (10.5) |
| Antineoplastic and Immunomodulating Agents | 35.1 (24.1) | 27.0 (20.6) | 13.9 (6.4) | 11.3 (13.7) | 12.0 (14.4) | 9.7 (4.0) | 8.0 (6.4) | 24.2 (26.5) |
| Antiparasitic Products, Insecticides and Repellents | 32.0 (21.7) | 31.4 (14.3) | 21.2 (15.8) | 38.4 (18.2) | 15.4 (10.8) | 11.5 (7.2) | 13.8 (9.9) | 26.1 (20.5) |
| Antivirals for Systemic Use | 23.3 (15.1) | 26.2 (11.5) | 11.7 (3.4) | 24.2 (33.9) | 10.0 (11.4) | 8.0 (0.1) | 8.0 (0.0) | 18.7 (16.2) |
| Blood and Blood Forming Organs | 12.2 (27.6) | 5.1 (6.5) | 5.9 (1.5) | 6.0 (1.5) | 6.0 (0.1) | 8.0 (2.2) | 8.0 (3.0) | 7.6 (4.0) |
| Cardiovascular System | 18.9 (5.1) | 16.0 (10.6) | 14.1 (10.7) | 9.0 (15.6) | 16.4 (9.5) | 12.0 (3.6) | 7.9 (3.1) | 12.2 (10.8) |
| Dermatologicals | 34.5 (33.3) | 21.4 (24.9) | 12.8 (7.9) | 10.0 (0.0) | 17.0 (7.0) | 11.5 (3.3) | 11.1 (11.2) | 16.9 (21.6) |
| Diagnostic drugs | 17.1 (12.8) | 19.7 (4.8) | 12.0 (11.4) | 13.7 (5.1) | 18.8 (8.8) | 11.3 (0.3) | 12.3 (5.2) | 14.3 (11.0) |
| Genito Urinary System and Sex Hormones | 41.0 (18.1) | 28.7 (58.2) | 32.6 (12.1) | 25.6 (13.3) | 24.9 (31.1) | 11.9 (2.8) | 7.5 (4.5) | 25.6 (29.0) |
| Musculo-Skeletal System | 15.9 (10.7) | 12.0 (7.6) | 6.9 (6.7) | 6.0 (3.9) | 9.9 (2.2) | 7.8 (5.2) | 8.0 (1.9) | 9.9 (7.4) |
| Nervous System | 20.7 (14.8) | 23.3 (25.7) | 12.0 (11.5) | 23.9 (4.9) | 10.0 (0.1) | 11.0 (16.4) | 12.0 (2.4) | 14.9 (13.3) |
| Other Antiinfectives for Systemic Use | 13.0 (15.1) | 7.4 (1.6) | - | 7.9 (2.1) | 21.8 (7.3) | 11.0 (0.0) | 8.0 (2.1) | 9.7 (8.2) |
| Respiratory System | 27.7 (12.9) | - | 12.2 (6.4) | 8.6 (9.2) | 17.4 (0.0) | 13.0 (5.1) | 12.0 (37.4) | 14.9 (11.0) |
| Sensory Organs | 14.2 (9.0) | 11.2 (0.0) | 6.0 (3.0) | 33.8 (12.9) | - | 10.9 (4.0) | 8.0 (2.1) | 10.9 (10.0) |
| Systemic Hormonal Preparations, Excluding Sex Hormones & Insulins | 12.3 (8.2) | 30.5 (23.9) | 13.8 (6.9) | 6.4 (24.8) | 19.2 (13.2) | 9.9 (26.2) | 12.0 (17.6) | 12.0 (21.7) |
| Standard Review Drugs | 33.5 (23.3) | 22.9 (20.5) | 16.2 (11.7) | 17.8 (19.0) | 13.0 (13.7) | 12.0 (3.7) | 12.0 (4.9) | 18.2 (19.9) |
| Priority Review Drugs | 21.6 (18.5) | 12.1 (14.8) | 6.4 (6.4) | 6.0 (3.1) | 7.1 (4.3) | 8.0 (4.9) | 8.0 (1.0) | 8.1 (9.0) |
| Total | 26.6 (23.6) | 18.5 (18.1) | 12.2 (11.7) | 10.0 (18.6) | 10.0 (10.8) | 10.6 (4.1) | 8.1 (4.2) | 12.0 (16.3) |

*.*
